# Common needs in uncommon conditions: a qualitative study to explore the need for care in pediatric patients with rare diseases

**DOI:** 10.1186/s13023-022-02305-w

**Published:** 2022-04-04

**Authors:** Rosanne M. Smits, Eline Vissers, Rosan te Pas, Noor Roebbers, Wout F. J. Feitz, Iris A. L. M. van Rooij, Ivo de Blaauw, Chris M. Verhaak

**Affiliations:** 1 Department of Medical Psychology, Amalia Children’s Hospital, Radboud University Medical Center, PO 9101, 6500 HB Nijmegen, The Netherlands; 2 Division of Pediatric Urology, Department of Urology, Radboudumc Amalia Children’s Hospital, Nijmegen, The Netherlands; 3 Department for Health Evidence, Radboud University Medical Center, Nijmegen, The Netherlands; 4 Department of Pediatric Surgery, Amalia Children’s Hospital, Radboud University Medical Center, Nijmegen, The Netherlands

**Keywords:** Rare disease, Complex condition, Psychological needs, Caregiver burden, Qualitative research, Quality of life, HRQoL

## Abstract

**Background:**

Challenges faced by children diagnosed with a rare disease or complex condition and their family members are often characterized by disease-specific complexities, such as a prolonged diagnostic process, an uncertain prognosis, and the absence of curative treatment. The psychological burden of living with a rare disease or complex condition is often understudied and may present overarching concepts that shape the general experience of having been diagnosed with a rare condition. The present study examines common needs from a comprehensive perspective combining relevant aspects from the rare disease literature in a theoretical perspective from pediatric psychology, such as a family-centred, developmental and interdisciplinary approach. An exploratory study was designed among parents from children with a rare disease or complex condition in an Integrated University Children’s Hospital in the Netherlands. Semi-structured interviews were conducted with open-ended questions based around the experience of having a child diagnosed with a rare condition, such as the psychosocial impact on the child and it’s development, the impact on the family, and how provided care was experienced.

**Results:**

Twelve interviews were analysed with a thematic content analysis to identify common needs. Eight themes followed from the analysis and uncovered the need for (1) family-focused care, (2) coping with uncertainty, (3) empathic communication, (4) practical support, (5) information, (6) psychological support, (7) interdisciplinary care, and (8) social support.

**Conclusions:**

The results from our study provide directions for research and health care to support young patients with a rare disease or complex condition and their families. Moreover, our results demonstrated that there are overarching concepts across different rare diseases that may be optimally supported with interdisciplinary care.

**Supplementary Information:**

The online version contains supplementary material available at 10.1186/s13023-022-02305-w.

## Introduction

Many rare diseases start in childhood and continue throughout the lifespan [1]. The criteria and definitions for rare diseases range differently worldwide. For example, a rare disease is defined by a prevalence of 1:2000 in the EU, and a prevalence of 1: ~ 1600 in the US (less than 200,000) [2, 3]. Despite these varying numbers, the World Health Organization has classified 5000 to 8000 rare diseases which mostly have a genetic origin and affect approximately 55 million Europeans and North Americans (WHO, 2013). Thus, albeit the inherent rarity of a rare disease, it is not rare to have a rare disease [3, 4]. From a medical care and research perspective, the rarity of a disease faces many challenges. Rare diseases are often studied within small samples and result in heterogeneous disease outcomes [5]. Consequently, rare diseases are characterized by limited information about prognoses and can involve a long diagnostic process [3, 5]. Additionally, in many cases there is no curative treatment available and patients have to manage the symptoms of their disease with uncertainty regarding its course [6, 7]. A complex condition is currently more difficult to define and suggestions for a definition include the involvement of multiple organ systems and related medical specialties [8], but faces many similar challenges as rare diseases. Overall, rare diseases and complex conditions ask for a specific health care approach. To support patients and their families it would be useful to identify overall patterns in the need for their specific interdisciplinary care.

Psychological impact and health-related quality of life (HRQoL) are topics that are understudied in the current literature of rare diseases, even though these topics may present overarching concepts that shape the general experience of having a rare disease. Quantitative data on HRQoL in patients with a rare disease often fail to properly incorporate the patient perspective by merely focusing on disease-related variables [9]. Moreover, there is a lack of studies that exploratively navigate the psychological impact on patients with a rare disease and their family members and the subsequent need for care [3]. Currently, there is an urgent call from international and national patient organizations to reduce psychosocial vulnerability in (genetic) rare diseases and complex conditions, which has been described in the RARE 2030 Foresight Study [10]. This study underlines the need to raise awareness for mental well-being in rare diseases. To tap into this momentum, it would be valuable to firstly scope the overarching needs and challenges faced across a broad spectrum of rare diseases and complex conditions, and subsequently adopt a disease-specific approach to follow up on these needs. For example, one of the few psychosocial studies that focused on rare diseases in children addressed the need for parental support across different rare diseases with an emphasis on social needs, informational needs and emotional needs [11]. This underlines that even though disease-specific management for patients may be heterogeneous, but that the supportive needs of parents and patients may be homogeneous. By further exploring these general themes across different types of rare diseases or complex conditions, more insights could be gained to improve care and support for patients and families affected by a rare condition.

Evaluating psychological needs in pediatric diseases requires a different approach from adult populations and need to be viewed from an integrative framework [12]. This ecological framework is elaborated extensively in the field of pediatric psychology [13]. Factors within this framework address (1) the role of family as a system, (2) examine the child’s development, and (3) assess the need for interdisciplinary care (e.g. from different medical and psychology disciplines). In a family-focused approach, the complex interplay between parents and siblings is considered and addresses the powerful reciprocal influence over the child’s adaptation to illness. The family’s ability to adequately adapt to a child’s illness and adhere to the treatment recommendations is therefore greatly determined by the psychosocial vulnerability of the family [12, 14, 15]. In a developmental approach, intervention programmes are adjusted to important transitional ages such as school starting age, when children start building social relationships, or when important cognitive developments take place [12]. Finally, studies that focus on the psychological burden of having a rare disease stress that interdisciplinary support or care may have a substantial impact on HRQoL, and is a frequently addressed need from patients and their families [12, 16–19]. Thus, when examining psychological needs in pediatric rare diseases or complex conditions research aims should include a family-centred, developmental, and interdisciplinary approach.

The present study explores common needs for patients and families with rare diseases or complex conditions. This study uses a qualitative approach and incorporates an integrative framework combining relevant aspects from the rare disease literature. In addition to previous rare disease literature, we aimed to explore these general themes across different types of rare diseases and complex conditions, to gain overarching insights and to aid optimized care and support for patients and families.

## Methods

### Study Design

The participants in this study took part in a larger study called ‘TRANSIT’, a cohort study designed to examine the impact of the child’s complex somatic disease and their parents’ experiences with the health care system. The study took place from January 2019 to December 2019. Participants were sent an invitation letter from the Department of Medical Psychology (Radboudumc, Nijmegen, The Netherlands) to take part in the study (N = 93). Sixteen participants enrolled in the study and started with a questionnaire (T1) to screen for psychosocial vulnerability in parents of patients (outside the scope of this study). After four months, the same participants were approach to follow-up with the interview part for the current study (T2). Ethical approval for the study was obtained from the ethics committee of the Radboudumc Nijmegen in the Netherlands (2018–4896). The interview questions were focused on the psychosocial impact of the disease on the child, the family, and the experiences with health care [12, 14, 15].

### Participants

Inclusion criteria were parents of children with a (genetic) rare disease or complex condition that have had a follow-up or treatment for their child less than one year ago, and were able to speak and understand Dutch. All participants were parents of registered patients at an Integrated University Children’s Hospital (Amalia Children’s Hospital, Radboudumc, Netherlands) for either regular check-ups or further treatment appointments and agreed to take part in this study. All interviews that were selected from the TRANSIT cohort were parents of children with a rare disease or complex condition. Participants provided written informed consent to be interviewed. After the interviews, participants were gratified with 20 euros for their participation.

### Procedure

Open-ended questions were used to identify concerns, problems or general experiences within the health care system and participant’s own environment. The interviews were conducted by trained researchers RTP and NR. The interviewers guided the topics, but left room for more elaboration from the participants and asked follow-up questions for further explanation when necessary. An overview of the interview questions is provided in Additional file 1. The interviews lasted between 60 to 90 min and were recorded with consent from participants after which they were transcribed verbatim.

### Data analysis

A thematic content analysis approach was used and a category system was developed that reflected central themes articulated by parents [20]. Researcher RMS and EV independently drafted themes from the transcripts of the interviews. RMS proposed the first category system, which was revised and rewritten by EV. Final categories were established through discussion. After the category system was established, the resulting system was discussed with a third researcher (CV) to reach agreement (see Additional file 2 for the category system).

## Results

### Demographic characteristics

Of the 16 parents approached, 12 participated in the interviews. Reasons for refusal/not participating were not being able to come in contact via e-mail or telephone [2], indication for psychological vulnerability [1], and reason unknown [1]. Mean age of the patients was 5.9 years (SD = 4.3). Diagnosis included anorectal malformations (ARM; n = 3), hypospadias (n = 2), epispadias (n = 1), therapy resistant complex incontinence (n = 1), ARX-related epileptic encephalopathy (n = 1), Turner syndrome (TS; n = 1), metabolic disorder (methylmaloic acidemia; n = 1), foetal alcohol syndrome (FAS; n = 1), and one patient without a diagnosis yet (n = 1, symptoms described in Table 1). Demographic characteristics are described in Table 1.

**Table 1:** Demographic characteristics of the 12 participating families

Diagnosis	ORPHA code	Age (child)	Family composition (x)	Interviewee
ARM^a^	96,346–557	1 year	M^b^F^c^, 3	M
Hypospadias	95,706	2 years	MF, 3	M
Complex incontinence^e^	N/A	7 years	MFS^d^, 4	M
ARX-related epileptic encephalopathy	182,079	9 years	MFS, 4	M
Undiagnosed^f^	N/A	7 years	MFSS, 5	M
ARM	96,346–557	10 months	MFS, 4	M
FAS^g^	1915	13 years	MF*, 3	F
ARM	96,346–557	6 years	MFSSS, 6	F
Epispadias	96,346	10 years	M, 2	M
Hypospadias	95,706	1.5 years	MFS, 4	M
Turner Syndrome	881	11 years	MFSS, 5	M
Methylmalonic acidemia (MMA)	289,504	2.5 years	M, 2	M

x = number of members of the family

^a^Anorectal malformation; ^b^M = mother; ^c^F = father; ^d^S = sibling; ^e^Complex Incontinence; therapy resistant urinary incontinence with the need for diapers; ^f^symptoms: fever seizures, reflux, rectal bleeding, speech and language developmental delay, house dust mite allergy, cryptorchidism; ^g^Foetal alcohol syndrome; *Adoptive parents

### Common needs in problems experienced

**Fig. 1:**
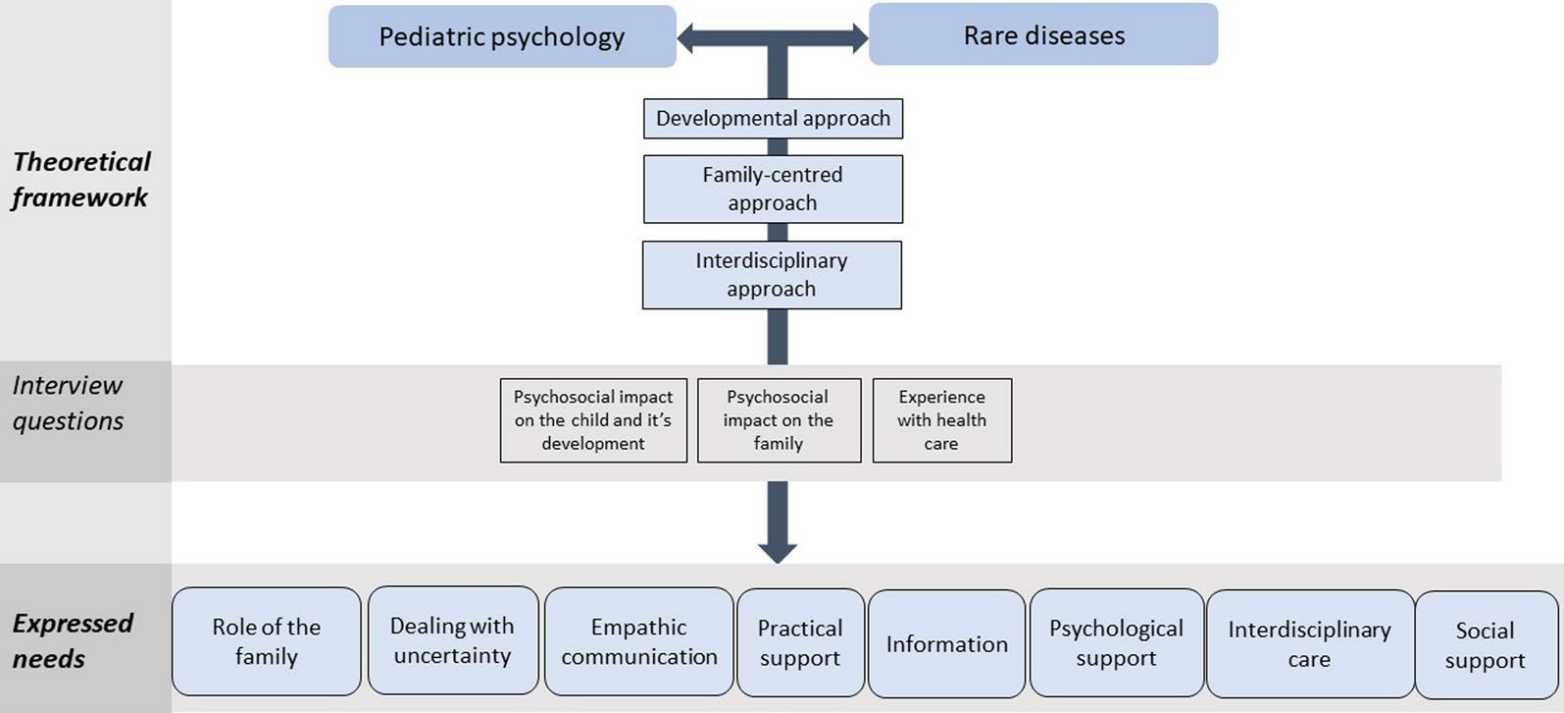
Conceptual overview of study design and results

Eight common needs were identified based on the answers from the interviews: (1) family-focused care, (2) coping with uncertainty, (3) empathic communication, (4) practical support, (5) information, (6) psychological support, (7) interdisciplinary care, and (8) social support (Table 2 and Fig. 1).

**Table 2:** Expressed needs addressed by the 12 participants

	Frequency (%)
1. Family-focused care	7 (58%)
2. Coping with uncertainty	6 (50%)
3. Empathic communication	4 (33%)
4. Practical support	6 (50%)
5. Information	3 (25%)
6. Psychological support	7 (58%)
7. Interdisciplinary care	6 (50%)
8. Social support	3 (25%)

#### Family-focused care

From the 12 interviewees, seven respondents expressed the need for more focus on the role of the family when asked about the psychosocial impact on the parents and the siblings of the child with a rare disease or complex condition. The attention for the role of the caregiver was sometimes overlooked in the eyes of the parents. They expressed concerns in managing the disease at home and the need for an interdisciplinary approach that also incorporates the family members and all aspects of the disease, both practical and emotional. Parents also expressed that it was important to realize how the disease of their child affected their relationship with other members of the family. For example, siblings of children with a rare disease experience a different kind of relationship with their parents because their sister or brother with a rare condition requires more attention from the parents. These answers illuminated the role of the family as a system that was consequently affected by the involved disease.

There should be more attention for the brothers and sisters. We arranged care for her brother ourselves, but I think there could be more initiative from the hospital for this.

Her brother, he also has to deal with it every day. He was also very independent at a young age because I often needed all the attention for his sister in the morning because she had seizures.

#### Coping with uncertainty

Six from the twelve interviewees addressed difficulties in coping with uncertainty. Parents expressed the feeling of uncertainty in a couple of areas. Firstly, the period before receiving a diagnosis of their child was accompanied with a considerable amount of uncertainty and a higher need for psychological support during that period.

The first weeks there was a lot of uncertainty, then the tension for the operation came.

These parents reported that not knowing what was wrong with and what was needed for their child was a very stressful period, which was followed by a late diagnosis and surgery that also brought a certain level of stress. Distress and anxiety was expressed in the weeks before the diagnosis in coping with uncertainty. Secondly, uncertainty was expressed in regards to why certain treatment procedures were executed or why treatment procedures were different from the ones used in other hospitals, which also brought stress to parents. Other points of uncertainty often referred to the future perspectives. This could pertain to the course of the disease and its trajectory, but also to the future of the child that includes insecurities in many areas of life. For example, parents expressed concerns related to the prognosis of the disease, which in the case of rare diseases often remains unclear.

With common conditions, there is a protocol for everything. Everything for us is less scientific and more ad hoc care. It sometimes feels vague, and we do not know what options we have, and what can be expected.

Other topics of concern focused on the child’s development, for example how the disease would affect certain life changes, such as puberty. These parents expressed concerns about making substantial life decisions for their child, uncertain about their child’s best interest.

We do worry about the future from time to time: will things always go well?

Parents also report a form of uncertainty that includes a lack of understanding of what is happening to siblings. This causes for anxiety and a need for parents to take great care in looking out for their other children.

#### Empathic communication

The need for empathic communication was expressed in four interviews. In some cases, the parents did not feel taken seriously or they felt like they were not being heard.

…it’s still personal. At first I did not feel heard.

In other cases, the parents felt the impact of the disease was trivialized. The parents felt unheard when it was expressed that they did not want to proceed with a specific treatment plan.

…I feel that the impact is sometimes downplayed. And I am mainly talking about the impact of drug changes where you get undesirable behavior.

Furthermore, communication about the needs of the parents sometimes seemed to be overlooked.

…In any case, there is no good communication about the expectations. For example what we can expect from hospitalization.

Parents wanted to be involved in medical decision and expressed the need to know why certain procedures were being conducted. Conversely, the parents expressed a need for more consideration and attention to their needs.

#### Practical support

Practical difficulties in terms of coordination with health care providers were mentioned in six interviews. Health care professionals contribute to the management of the disease by coordinating health care and the communication between health care providers. However, some parents felt that health care providers cooperated as separate islands, and miscommunication often occurred.

When things were not going so well, it would take a while to get in contact with the right person. This was very stressful at the time.

Moreover, parents reported that caring for a child with a rare disease requires a high level of flexibility and a continued requirement of attention in managing the disease. For example, everyday life (e.g. specific dietary needs, babysitting, work-life balance) can be more complex for children with a rare disease.

He has to follow a strict diet that takes one day extra for meal preparing.

We have to be flexible in many aspects. For example, we cannot just hire a ‘normal’ sitter.

I had to arrange a workplan together with a social worker to figure out how I could maintain my job and care for my child.

#### Information

Informational needs were voiced in three interviews. For example, parents stated that they wanted more information about why their child received another procedure than a child with the same condition they knew. Another example is that parents expressed that they wish they would have received more information about the treatment procedures and after care.

We heard that someone we know has a child with the same condition but received different treatment in another hospital. When we asked about this, we didn’t receive a clear answer why this treatment was different. This raised a lot of questions to us.

However, often participants clearly stated that the possibility of asking questions to health care professionals was generally satisfactory and effective. In addition, as a suggestion one of the participants expressed the need of a written checklist from the doctors with clear instructions and explanations on the treatment plan of their child. This would have helped to gain more insights on the disease and have an overview of the material they needed to know.

We would have liked it if we had received some sort of checklist. A bit more clarity and on paper I think.

The concern was that going to the hospital, families often saw many different health care professionals due to changing shifts, which sometimes led to the health care professionals explaining things differently. This made it desirable to have an overview on paper for parents.

Moreover, most parents said to have looked at the website of their hospital, explaining the disease of their child but many of them did not use the information or did not find the information about the psychosocial impact of the disease on the child and the family. Lastly, as has already been mentioned under uncertainty, parents were sometimes in need of more explanation or information on the treatment procedures and actions conducted by the health care professionals.

#### Psychological support

In seven interviews, it was clear that psychological support was either necessary for the child itself or for one of the family members. Moreover, it was expressed that parents found it difficult to ask help for themselves as they prioritized the needs and support of their child above their own.

Now, a year later, I am seeing a psychologist myself. I was very much in survival mode during that period before the surgery. I was very scared and insecure during that period. Especially because I couldn't handle the care for my daughter.

Siblings of children with a rare disease can also be in need of psychological support to cope with the situation, to understand it or to express their fears.

Yes, at the beginning he was very scared [for his brother] that things were going pretty bad for him and that he was afraid of losing him.

In one case parents expressed that no psychological support was offered, even though the mother clearly would have benefited from the support, as she reported so herself.

#### Interdisciplinary care

A large theme across participants was the need for a more interdisciplinary approach which was expressed by half of the interviewees. Some parents suggested that an interdisciplinary team of health care providers could have been involved at an early stage, for example before the diagnosis when psychosocial support was specifically needed.

In hindsight it would have been very helpful if an interdisciplinary team was involved from the start. For example with a psychologist, social working, and physiotherapist.

Alternatively, one of the interviewees stated that she sometimes experienced ‘too much interdisciplinary care’.

Especially for the first time, there were three strange adults in the room [at least one doctor and one psychologist], that was too much for X and he was too afraid to say anything.

The communication between peripheral hospitals and expertise centres like Radboudumc were also mentioned by parents. In one case, the transmission of information and medical files went very well, and doctors of the peripheral hospital were well aware of the trajectory that parents were following in expertise centres. In another case, parents had a separate psychologist that was not connected to an expertise centre and were unable to communicate their needs and wishes with the treating physician. Other participants also voiced the need of good coordination of files and information between different health care professionals.

I found it very hard to tell my story again to a different psychologist. I wish this was coordinated better between the hospital and practice, so that I didn’t need to relive those moments again.

#### Social support

In three interviews parents explained that they felt a lack of social support, or they did not feel understood by their social networks. For example, when their concerns were not understood or when they did not feel taken seriously by their social networks.

Not everyone understands that it can still be stressful to go to the hospital. Even when it is for check-ups: They forget my worries most of the time.

In my opinion, his grandmother does not understand X. My sister too, by the way. They think everything is okay and I feel they do not respond seriously to my worries.

Another parent stated that caring for her child consumed so much of her time, and that she often finds herself feeling socially isolated.

A social life requires extra attention because you are so busy with caring for your child. I feel sometimes that my friends and family forget that, when I don’t talk about it with them.

## Discussion

The present study examined common needs as they were mentioned by parents of children with various types of rare diseases or complex conditions with a qualitative research method. In addition to the scarce literature available on this topic, the current study approached the need for care from a theoretical pediatric perspective that specifically addressed the role of the family as a system, considers the child’s development, and assesses the need for interdisciplinary care. These topics involve important aspects from pediatric psychology and rare diseases literature [2, 9, 11, 12, 14, 21]. Results from our study brought forth eight common needs in pediatric patients with a rare disease or complex condition: (1) family-focused care, (2) coping with uncertainty, (3) empathic communication, (4) practical support, (5) information, (6) psychological support, (7) interdisciplinary care, and (8) social support.

The first common need that was addressed in the majority of our sample (7 out of 12 interviewees) mentioned a lack of support in care for the role of the family, for example the need for practical support, such as managing the disease at home. Another need frequently mentioned was the need to incorporate the impact on the family and the relationship with siblings in the care for these families. In addition, addressing the psychosocial well-being of siblings is increasingly gaining attention as a recent study provides evidence that siblings of children with rare disorders display more psychosocial problems than controls [22]. These findings underline the importance to consider the family as a system in pediatric care, and validates the influence of the child’s family to adapt to illness [23–25].

A second need commonly expressed was dealing with uncertainty. In the interviews, uncertainty was expressed towards different aspects of having a rare disease or complex condition, such as dealing with uncertainty during the diagnostic process, dealing with uncertainty regarding treatment, prognosis, and the future, and communication about uncertainty to the rest of the family. Dealing with medical uncertainty in general, aside from uncertainty impacted by rare diseases, is a concept widely studied in health care. Different aspects of uncertainty have been underlined in a three-dimensional model of uncertainty in health care and a revised model for uncertainty in health care settings [26, 27]. These models address three broad categories of uncertainty: scientific, practical, and personal uncertainty. The different types of uncertainty mentioned in the present study fit well to these models, but leave room for specific types of uncertainty that are characteristic for rare diseases (such as the absence of curative treatment possibilities and lack of peer support) and the role of uncertainty for the family as a system (such as communication about uncertainty to siblings), a common theme in pediatric medical psychology. These concepts could be further explored and future research and specific care programs should focus on coping with uncertainty, as this is an insuperable theme in rare diseases, for example with acceptance and commitment therapy [28].

Two other needs that were mentioned are closely related to uncertainty, namely the need for empathic communication and for information. Interviewees expressed the need to be taken seriously and the need for shared decision making. Moreover, there was a need for the possibility to ask questions, and for information on emotional coping. In light of previous literature, these needs are commonly expressed in adult and pediatric patients with a rare disease as patients or parents often become experts of their own diagnosis and feel frustrated about professionals’ lack of understanding and the impact on the quality and access to care [1–3].

The need for interdisciplinary care was also frequently mentioned. This was stated on its own, but was also expressed in the need for practical support and for psychological support. Interviewees mentioned their need for coordinated care and coping with burdensome care. In addition, emotional coping with a rare disease was mentioned, which was subdivided in the need for care for the child and the family/parents. Another important psychological need focused on parental procedural distress, for example with invasive procedures that can be emotionally burdensome for parents to execute (i.e. dilating for children with anorectal malformations). This is commonly expressed in the field of pediatric psychology and stress the need to study parenting behaviour interventions that reduce pediatric procedural distress [29, 30]. Another need that is commonly expressed in children with rare diseases is the need to involve an interdisciplinary approach when transitioning to adult care because this is a pivotal moment in social, physical, and sexual development. Even though this need was not addressed in the current sample (all children were 10 years or younger), this is a frequently addressed concern and therefore worth mentioning [16]. Finally, the need for social support was expressed by several interviewees as they felt a lack of support and understanding from their family, friends or social networks.

To interpret the common needs mentioned in the present study, several considerations have to be taken into account. First, the present findings primarily looked for needs and insights where care for rare diseases can be optimized for families. Therefore it is important to note that, besides the eight needs that were pointed out, the participants overall felt like their needs were being met satisfactorily by the hospital. Second, it is important to note that the participants and patients from this study received care in an academic medical centre (Radboudumc, The Netherlands). This brings us to another consideration, namely that the medical centre in which the study was conducted is an acknowledged expertise centre for a broad range of (pediatric) rare disease, and therefore experiences may be more positive than in more unspecialized hospitals. Another topic of reflection is the categorization of the eight needs that was provided. As we aimed to conceptualize a comprehensive overview of the outcomes, it is worthy to note that overlap between the common needs exist and have to be interpreted from a holistic framework, rather than isolated concepts. For example, psychological support was a recurrent need that overlapped in several common needs (i.e. the need for interdisciplinary care, attention for the role of the family, dealing with uncertainty). Lastly, we acknowledge limitations from the small sample size in this study. Even though efforts were made to include a broad range of rare diseases in the study with eight diagnoses and one undiagnosed patient, this may still be a small range of existing rare diseases. However, most needs that were addressed exposed an overlap in needs expressed in the current study, and needs addressed in previous literature with rare (pediatric) diseases [2, 3, 6, 9, 11, 16, 21].

To conclude, the findings from the current study shed light on general needs that play an important role in the psychological impact of having a child with a rare disease or complex condition. To add to current literature, we integrated important concepts from the field of pediatric medical psychology (family-centred and developmental approach) and the field of rare diseases (interdisciplinary approach) in our interview questions, thereby providing additional insights in the support for pediatric patients with rare conditions. Additionally, the findings of our study may serve as a foundation to connect the needs in pediatric rare diseases with evidence-based interventions to support these needs. To extend the results of this study, future research aims should focus on evaluating existing psychosocial interventions that may be suitable to support the needs of families faced by rare disease. An important solution to this could be by involving patient networks in tailoring such interventions to the general needs of patients and parents with rare diseases, thereby encouraging co-creation. For example interventions that address the family as a whole (i.e. comprehensive behavioral family lifestyle interventions (CBFLIs)), or interventions that support parents in procedural distress (i.e. behavioral treatment for fecal incontinence or distraction for needle pain), or coping with uncertainty could be suitable to support the common needs in uncommon conditions [28, 29, 31].

## Supplementary Information

**Additional file 1.** Open-ended interview questions.

**Additional file 2.** Category system.

## Data Availability

The datasets generated during the current study are available from the corresponding author on reasonable request.
