# Supplementary material for: Common needs in uncommon conditions: a qualitative study to explore the need for care in pediatric patients with rare diseases
Source: Orphanet J Rare Dis. 2022 Apr 4;17:153. doi: 10.1186/s13023-022-02305-w (PMC8981675; doi:10.1186/s13023-022-02305-w)
Supplement: Supplementary file 1 — Additional file 1. Open-ended interview questions. [file 13023_2022_2305_MOESM1_ESM.docx]

**Supplementary File I: Open-ended interview questions**

**Introduction**This interview is part of the TRANSIT study. The TRANSIT project aims to improve the organization of psychosocial care and to enable care providers from different organizations involved in the care of the child to work well together, across the walls of their own organization. The focus is to optimize health care that meets the needs and wishes of the child and family.

In this interview we ask questions about the care in and outside of the hospital. We would like to focus primarily on the psychosocial support and care that you have received in addition to medical care. The interview will last approximately 60 to 90 minutes. This conversation is about your experiences with care and your needs. There are no right or wrong answers.

Your answers will be processed anonymously. Results are used to answer the research questions as stated in the information letter. We would like to record the interview. This helps us work it out. The recording will be treated confidentially and analyzed anonymously.

**Psychosocial screening**• How did you feel about completing a screening list about psychosocial risks?
• How were the results reported back to you?
• What did the results bring you?
• Which follow-up actions have taken place from the hospital in response to the results?

**Ask for help and needs**• How have the needs of your child and the family been considered?
• How did you experience this?
• What did you miss in this?
• What was good about asking your child's and family's needs?
• What did asking about your needs giveyou?
• What follow-up actions have been taken from the hospital regarding the needs of your child and the family?

**Care plan**• What did you think of a care plan being drawn up?
• In what way were you able to contribute to this?
• What did you miss in the care plan?
• What was good about the care plan?
• How did you use this care plan?
• What did the care plan provide for your child and the family?

**Information psychosocial impact**• What did you think of the information on psychosocial problems on the Amalia Children's Hospital website?
• How did you find this information?
• How did reading this information affect you?
• What did you miss in this information?
• What did this information mean to you?

**Psychosocial care in the hospital**• Which healthcare providers did you have contact with in the hospital?
• How have they supported you with emotional, social and practical problems?
• How did you experience this?
• What would you have liked to see different in this support?
• What would any adjustments in support do to your child and the family?

**From hospital to home**• What psychosocial support does your child and family need (or have had) at home?
• How did the hospital help to realize this?
• What helped you to arrange support?
• What do you think could have been done better?

**Care at home**• How do you experience psychosocial support in your home situation?
• How is the communication between the different care providers?
• How do they use the care plan that has been drawn up?
• How do you use the care plan at home?
• What is your own role as a parent? How do you feel about that?
• If you had questions, what did you do?
• What do you miss in home care?
• What would help you cope better with the situation at home?
