# Supplementary material for: Common needs in uncommon conditions: a qualitative study to explore the need for care in pediatric patients with rare diseases
Source: Orphanet J Rare Dis. 2022 Apr 4;17:153. doi: 10.1186/s13023-022-02305-w (PMC8981675; doi:10.1186/s13023-022-02305-w)
Supplement: Supplementary file 2 — Additional file 2. Category system. [file 13023_2022_2305_MOESM2_ESM.docx]

**Supplementary File II: Category system**

*The first category system was proposed by RMS and captured the need for information, the need for empathic communication from health care, the need for practical support, the need for a holistic approach, the need for psychological support, the need for the role of the caregiver, the need for a family-centered approach, and the need to cope with uncertainty. The categories were then revised and rewritten by EV to optimally capture the transcripts and a final category system was established in agreement with CV.*

1. **First proposed categories by RMS**

- Need for information
- Empathic communication from healthcare
- Practical support health care
- Holistic approach
- Psychological support
- Role of caregiver
- Family-centered approach
- Uncertainty

1. **Revised categories by EV**

- Family-focused care
- Coping with uncertainty
- Empathic communication
- Practical support
- Information
- Psychological support
- Interdisciplinary care
- Social support

1. **Final categories by RMS, EV, and CV**

- Family-focused care
- Coping with uncertainty
- Empathic communication
- Practical support
- Information
- Psychological support
- Interdisciplinary care
- Social support
